# Resting State Vagally-Mediated Heart Rate Variability Is Associated With Neural Activity During Explicit Emotion Regulation

**DOI:** 10.3389/fnins.2018.00794

**Published:** 2018-11-05

**Authors:** Elisa C. K. Steinfurth, Julia Wendt, Fay Geisler, Alfons O. Hamm, Julian F. Thayer, Julian Koenig

**Affiliations:** ^1^Department of Psychology, University of Greifswald, Greifswald, Germany; ^2^Department of Psychology, The Ohio State University, Columbus, OH, United States; ^3^Section for Translational Psychobiology in Child and Adolescent Psychiatry, Department of Child and Adolescent Psychiatry, Centre for Psychosocial Medicine, University of Heidelberg, Heidelberg, Germany; ^4^University Hospital of Child and Adolescent Psychiatry and Psychotherapy, University of Bern, Bern, Switzerland

**Keywords:** prefrontal cortex, amygdala, heart rate variability, emotion regulation, vagal

## Abstract

Resting state vagally mediated heart rate variability (vmHRV) is related to difficulties in emotion regulation (ER). The prefrontal cortex (PFC) provides inhibitory control over the amygdala during ER. Previous studies linked vmHRV with activity in the ventromedial PFC (vmPFC) during *implicit* ER. To date no study examined the relation between vmHRV and brain activity during *explicit* ER. vmHRV was measured during a 7 min baseline at T1 2–5 days preceding T2. At T2 *n* = 24 participants (50% female, *M*_age_ = 24.6 years) viewed neutral or emotional pictures of pleasant or unpleasant valence and were instructed to intensify or to reduce their present emotion using two ER strategies (*reappraisal* and *response* *modulation*) or to passively view the picture. Participants rated the valence of their emotional state from pleasant to unpleasant after ER. Whole-brain fMRI data were collected using a 1.5-T-scanner. We observed an association between resting state vmHRV and brain activation in the PFC and the amygdala during ER of unpleasant emotions. Groups based on vmHRV showed significant differences in the modulation of amygdala activity as a function of ER strategy. In participants with *high* vmHRV amygdala activity was modulated only when using *reappraisal* and for *low* vmHRV participants only when using *response modulation*. Similar, dorsomedial PFC activity in *high* vmHRV participants was increased when using *reappraisal* and in *low* vmHRV participants when using *response modulation* to regulate unpleasant emotions. These results suggest that individuals with *low* vmHRV might have difficulties in recruiting prefrontal brain areas necessary for the modulation of amygdala activity during explicit ER.

## Introduction

Emotion regulation (ER) can be defined as “*the evocation of thoughts or behaviors that influence which emotions people have, when people have them, and how people experience or express these emotions*” (Gross, 1998b). A host of brain imaging studies suggest that increased activation of the prefrontal cortex (PFC) is essential for ER (Beauregard et al., 2001; Ochsner and Gross, 2005; Wager et al., 2008). The PFC is theorized to have an inhibitory top down control over the amygdala during *explicit* (intentional) ER (Ochsner et al., 2002; Urry et al., 2006).

Resting state vagally-mediated heart rate variability (vmHRV), reflecting the potential for fast parasympathetic modulation of autonomic control of the heart, is inversely related to affective instability in daily life (Koval et al., 2013), and inversely correlated with self-reports on difficulties in ER (Berna et al., 2014; Beauchaine, 2015; Williams et al., 2015). Recent research suggests a relationship between brain activity and vmHRV, as higher resting state vmHRV is associated with stronger functional connectivity between the amygdala and the medial PFC (mPFC) across younger and older adults (Sakaki et al., 2016).

Importantly, in a series of studies on *implicit* (non-intentional) ER we have recently shown that (1) activity in ER related areas of the brain was positively correlated with coincident vmHRV when processing stimuli with its emotional significance in the attentional background (Lane et al., 2013); (2) that these relationships were absent in depression but increased and were similar to non-depressed controls after 12 week treatment with sertraline (Smith et al., 2014); and (3) that mPFC connectivity with the pons was negatively associated with depressive symptoms and positivity associated with coincident vmHRV when processing emotional stimuli with a non-emotional focus (Smith et al., 2015). These studies suggest that vmHRV and brain activity co-vary during *implicit* ER and that individual differences exist such that these associations are reduced or absent in persons with *low* vmHRV such as depressed patients (Kemp et al., 2010; Koenig et al., 2016).

While previous neuroimaging studies (e.g., Lane et al., 2013) demonstrated activity in the ventromedial PFC (vmPFC) during *implicit* (non-intentional) ER to be related to resting state vmHRV, the majority of neuroimaging studies on ER focused on *explicit* ER (i.e., intentional changes in affective state). However, surprisingly to date no study has examined the relationship between individual differences in resting vmHRV and brain activity during *explicit* ER.

Thus, the present study aimed to investigate the neural concomitants of *explicit* ER as a function of resting state vmHRV. Participants were trained in two ER strategies: *reappraisal* and *response modulation* and the neural activation during up- and down-regulation of emotions were measured. Resting state vmHRV was used to stratify participants into two groups. Based on previous findings it was hypothesized that individuals with *high* resting state vmHRV would exhibit differentiated activation of prefrontal brain regions and the amygdala according to the ER direction and that *low* resting state vmHRV would be related to less involvement of PFC structures and less modulation of the activation of the amygdala.

## Materials and Methods^1^

### Participants and General Procedures

Twenty-seven students participated in this investigation. Participants were selected if they were right-handed, had no current or prior mental disorders, and did not meet any MRI exclusion criteria, including metal in the body, claustrophobia, or pregnancy. Two participants did not complete the study due to schedule difficulties and one examination was discontinued due to technical problems. The final sample (*n* = 24) consisted of 12 female and 12 male participants, with a mean age of 24.6 years (range: 21–33). Participants received course credits or were paid an expense allowance of seven Euro. All participants gave written informed consent to the experiment approved by the University of Greifswald ethics committee. All procedures complied with the Declaration of Helsinki.

The investigation consisted of two parts: (1) a training session in the psychophysiological laboratory of the Institute of Psychology, 2–5 days prior to the experiment, and (2) the fMRI-experiment in the university clinic. During the training session participants practiced the two ER strategies and got familiar with the design of the study. Furthermore, their vmHRV was measured for 7 min at 1000 Hz in silence with a link belt, the Polar F5 Heart Rate Monitor Watch (Polar Electro Oy, Finland).

The fMRI-Session started with an introduction of the MRI-scanner and a medical check for participation by the clinic staff. The instructions for the ER strategies were repeated for each regulation direction and participants were asked to view all pictures attentively the whole time and not to close their eyes or move their head away from the picture. Pictures were projected on a tilted mirror mounted on the head coil.

### Stimulus Material

Based on normative valence and arousal ratings and the general content, two comparable sets of 36 pleasant and 36 unpleasant pictures were selected from the International Affective Picture System (IAPS; Lang et al., 2005). Arousal ratings were balanced for pleasant (*M* = 5.60, *SD* = 0.99) and unpleasant (*M* = 5.83, *SD* = 0.69) stimuli. Two comparable sets of 12 neutral pictures were used as reference stimuli. For each participant, the two picture sets were randomly assigned to the two ER strategies and were presented in six pseudo-randomized orders across participants^2^.

To standardize the regulation of emotions, specific instructions were given for each strategy. Generally, participants were instructed not to replace one emotion by another but to intensify (*increase*) or to reduce (*decrease*) their present emotion or to just view the picture passively (*maintain*; cf. Ochsner and Gross, 2005). Participants were instructed to apply two ER strategies: *Reappraisal* and *response modulation*. *Reappraisal* involved the cognitive variation of the dimension distance-intimacy. To increase the emotion participants were instructed to reduce the distance to the depicted content by imagining to be either personally involved in the scene or indirectly via persons to whom they have a close relationship (e.g., friends, family, etc.). To decrease the emotion, distance was enhanced by imagining the picture as a simulation or by imagining being a casual bystander. *Response modulation* focused on changes of respiration, body tension and facial expressions, which were intensified to increase and reduced to decrease the emotion. Participants were also instructed to modulate the visibility of their emotions according to the regulation goal. In the increase condition a possible spectator should recognize the experienced emotion, whereas in the decrease condition a possible spectator should not be able to recognize which emotion is experienced.

Participants rated the valence of their emotional state from pleasant to unpleasant (range 1–5) after regulating their emotions using the Self-Assessment Manikin (SAM; Bradley and Lang, 1994). Since these ratings were recorded after each ER period, they can be considered as indicators of regulation success.

### Experimental Paradigm

The experimental paradigm was a modified version from Eippert and co-workers (Eippert et al., 2007). It consisted of two experimental runs, one for each ER strategy. The strategy to be used first was balanced between participants. In each vmHRV group half (*n* = 6) of the participants started with *reappraisal* and the other half with *response modulation*. Every run was composed of 84 trials, 12 for each condition. The seven conditions reflected the seven regulation directions: pleasant-increase, pleasant-maintain, pleasant-decrease, neutral-maintain, unpleasant-increase, unpleasant-maintain, and unpleasant-decrease. All trials consisted of the following phases: induction, instruction, regulation and rating. Each trial began with the presentation of a picture for 2.5 s, which participants were instructed to view and to allow their emotional reactions to occur (induction phase). Then the instruction – equal sign (*maintain*), arrow up (*increase*), or arrow down (*decrease*) – appeared in the center of the picture, signaling the participants to regulate their emotions according to the practiced ER strategies during the training session. After 500 ms the instruction disappeared and the following 6 s were given for regulation with the picture still present. After the regulation phase SAM valence and arousal rating scales were presented for 3 s each and participants rated their current emotional experience. During an inter-trial-interval (8–10 s) a fixation cross was presented that signaled the participants to rest.

### fMRI-Data Acquisition

Whole-brain fMRI data were collected using a 1.5 T scanner Magnetom Symphony system (Siemens) equipped with an 8-channel head coil. During the two regulation runs 506 functional T2^∗^-weighted images were acquired in transversal direction using echo-planar imaging (EPI) with a repetition time (TR) of 4 s, Field of view (FoV) = 192 mm, matrix = 128 × 128, flip angle = 90°, echo time (TE) = 38 ms). Each functional volume comprised 33 slices (voxel sixe: 3 × 1.5 × 1.5 mm). Between the two functional runs, a high-resolution anatomical T1-weighted scan was acquired with a TR of 11 ms (FoV = 256 mm, matrix = 256 × 256, TE = 5.2 ms, voxel size: 1 × 1 × 1 mm).

### Data Analysis

**FIGURE 1:**
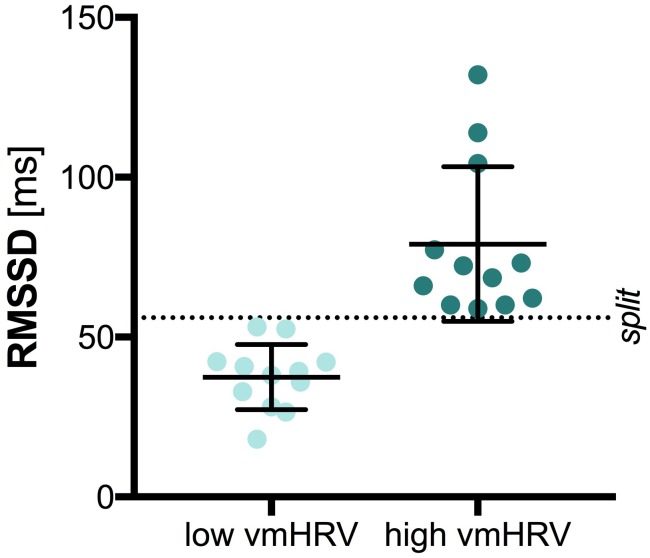
Vagally-mediated Heart Rate Variability by Group. Bars represent the mean and standard deviation. Dots represent individual participant data on the root mean square of successive differences (RMSSD) in milliseconds (ms); split: median split.

The central 5 min of the recorded 7 min HR data were analyzed with HRV Analysis (Niskanen et al., 2004). The root mean square of successive differences (RMSSD) is thought to represent vmHRV and was used as time-domain measure of vmHRV (Task Force of the European Society of Cardiology and Task Force of the European Society of Cardiology, 1996). The RMSSD in milliseconds (ms) was extracted for each participant and further included in analysis with SPSS (SPSS IBM Statistics 22). *High* and *low* vmHRV groups were separated on basis of the median split (*MD* = 56.05; *low* vmHRV *M* = 37.49; *high* vmHRV *M* = 79.08), as illustrated in Figure 1. Groups did not differ on age [t_(22)_ = 0.472, *p =* 0.642] or sex [χ_(1)_ = 2.667, *p =* 0.102]. Further, there was no significant difference between groups on mean heart rate [*low* vmHRV: *M* = 74.26 bpm (11.11); *high* vmHRV: *M* = 67.89 bpm (7.93): t_(22)_ = 1.62, *p =* 0.120]. Mean age (SD) was 25.3 (3.2) years in the *low* vmHRV group and 24.8 (1.9) in the *high* vmHRV group. Sex distribution was 4 women, 8 men in the *low* vmHRV and 8 women, 4 men in the *high* vmHRV group.

Valence ratings obtained after the regulation process were analyzed with a repeated measures analysis of variance (ANOVA) with the within-factors Strategy (*reappraisal* vs. *response modulation*), Valence (*pleasant* vs. *unpleasant*), and Direction (*increase* vs. *maintain* vs. *decrease*), and the between-factor vmHRV (*low* vs. *high*). Reporting of findings focusses on model fits and planned contrasts, adjusting for multiple testing using Bonferroni correction. Full reporting is provided in the Supplementary Table S1. All data were analyzed with SPSS (SPSS IBM Statistics 22). All results reported met a significance level of *p* < 0.05, unless otherwise noted. In case of violation of sphericity Greenhouse-Geisser corrected *p*-values are reported. Partial eta-squared ($\eta_{p}^{2}$) was used as measure of effect size, indicating the proportion of the total variance in a dependent variable explained by an independent variable while the effects of other independent variables and interactions are partialled out.

MRI-data pre-processing and statistical data analysis were performed with Statistical Parametric Mapping software (SPM8, Functional Imaging Laboratory, Wellcome Trust Centre for Neuroimaging, London, United Kingdom). The functional images of each subject were acquisition time corrected to the middle slice, realigned, co-registered to the anatomical image, segmented, and spatially normalized to a standard template of the Montreal Neurological Institute (MNI) and smoothed (FWHM 8 mm).

During first level analyses, for each participant a general linear model, as implemented in SPM8, was applied to the time-course of each voxel. The induction and regulation phase were modeled together using a boxcar function with a length of 9 s convolved with the hemodynamic responses function. The six movement parameters estimated during the realignment procedure were introduced as covariates into the model to control for variance caused by head movements (Johnstone et al., 2006). The resulting beta images were further analyzed on the second level in a full factorial model with the factors Strategy (*reappraisal* vs. *response modulation*), Valence (*pleasant* vs. *unpleasant*), and Direction (*increase* vs. *maintain* vs. *decrease*). The amygdala response to the experimental manipulation was quantified by means of the main effect of regulation direction. The prefrontal regions involved in ER were identified by means of the contrast ‘regulate (*increase* and *decrease*) vs. *maintain*.’

The time course of amygdala and prefrontal clusters exceeding a significance threshold of *p* < 0.001 (uncorrected) and an extend threshold of k = 5 were extracted using rfxplot (Gläscher, 2009) with spheres of a 3 mm radius centered around the individual peak activation within that cluster. Extracted scores were averaged for the 6 s of instructed ER and analyzed with SPSS (SPSS IBM Statistics 22) as described for valence ratings.

## Results

### Valence Ratings

**FIGURE 2:**
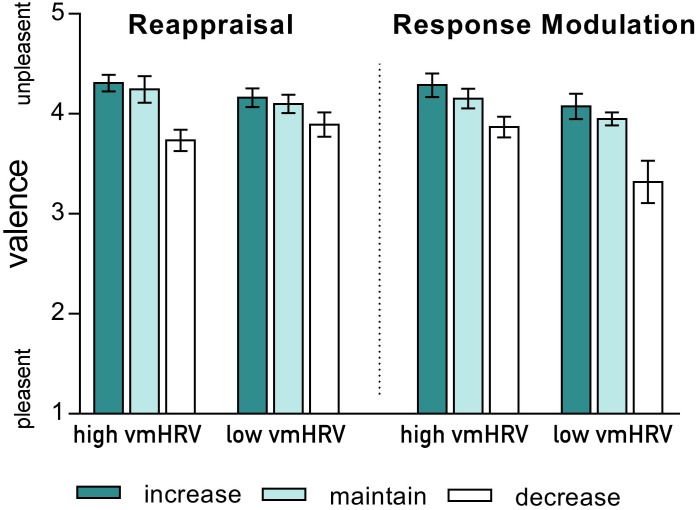
Valence ratings of the current emotional state after regulating emotions evoked by unpleasant pictures using either reappraisal (left) or response modulation (right) in participants with high and low resting state vagally mediated heart rate variability (vmHRV). Bars represent group means with standard errors.

As indicated by a significant 4-way interaction [Strategy × Valence × Direction × vmHRV: *F*_(2,44)_: 4.77, *p* = 0.021, η^2^_p_ = 0.18], the valence ratings of the participants current emotional state when using different ER strategies were influenced by vmHRV only when down-regulating unpleasant emotions. Compared to the corresponding maintain condition, participants with *high* vmHRV reported less unpleasant feelings after down-regulating their emotions using reappraisal [*t*_(11)_ = 5.22, *p* < 0.001 for reappraisal; *t*_(11)_ = 2.51, *ns* after bonferroni correction for response modulation]. In contrast, participants with *low* vmHRV reported a greater subjective reduction in unpleasantness when using response modulation [*t*_(11)_ = 3.28, *p* = 0.007 for response modulation; *t*_(11)_ = 2.25, *ns* after bonferroni correction for reappraisal; Strategy × Direction x vmHRV (*unpleasant*): F(2,44) = 5.49, *p* = 0.007, 0.20; see Figure 2]. No such effect of vmHRV was found for valence ratings after regulating emotions evoked by pleasant pictures (Strategy × Direction × HRV (*pleasant*): F < 1).

### Amygdala

Whole-brain analysis revealed a main effect of ER direction in clusters in both the right (MNI: x = 24, y = 4, z = -16; *F* = 7.36, *p_uncorr_* < 0.001, k_E_ = 5) and the left (MNI: x = -24, y = 2, z = -18; *F* = 12.51, *p_uncorr_* < 0.001, k_E_ = 15) amygdala.

As for valence ratings, resting vmHRV levels influenced right amygdala responses as a function of valence and regulation strategy (Strategy x Valence x Direction x vmHRV: F_(2,44)_: 3.11, *p* = 0.06, $\eta_{p}^{2}$ = 0.12). That is, the differentiation, between increasing and decreasing their negative states was more pronounced for *high* vmHRV participants when using reappraisal (increase > decrease; *t*_(11)_ = 3.95, *p* = 0.002) but not when using response modulation (*t*_(11)_ = 0.21, *ns*) and for *low* vmHRV participants when using response modulation (*t*_(11)_ = 4.57, *p* = 0.001) but not when using reappraisal (*t*_(11)_ = 0.95, *ns*). No effects of resting vmHRV were found on right amygdala response during the regulation of emotions evoked by pleasant pictures (Direction x vmHRV: *F*_(2,44)_ = 1.58, n.s.; Strategy x Direction x vmHRV: *F* < 1).

Resting vmHRV did not affect left amygdala responses during emotion regulation (Direction x vmHRV: F < 1), neither as a function of stimulus valence (Valence x Direction x vmHRV: F_(2,44)_ = 2.29, *ns*) nor as a function of regulation strategy (Strategy x Direction x vmHRV: F_(2,44)_ = 1.01, *ns*) or both (Strategy × Valence × Direction × vmHRV: F < 1).

### Prefrontal Cortex

Whole-brain analysis revealed five clusters in the dorsolateral PFC and one cluster in the dorsomedial PFC showing a more pronounced response to conditions in which participants were instructed to regulate their emotions (Regulate > Maintain; see Table 1).

**Table 1:** Prefrontal clusters showing more pronounced activity during regulation conditions (increasing and decreasing emotions) compared to maintain conditions.

No	Region	Side	MNI-coordinates			k_E_	*t*-score^∗^
			x	Y	z		
1.	dorsolateral	L	-20	50	30	11	3.80
2.		L	-26	52	24	16	3.68
3.		L	-56	22	8	5	3.42
4.		R	20	38	36	27	3.74
5.		R	20	24	18	5	3.53
1.	dorsomedial	R	12	46	36	8	3.26


*p_*uncorr*_ < 0.001; MNI, Montreal Neurological Institute; k_*E*_, cluster size.*

**FIGURE 3:**
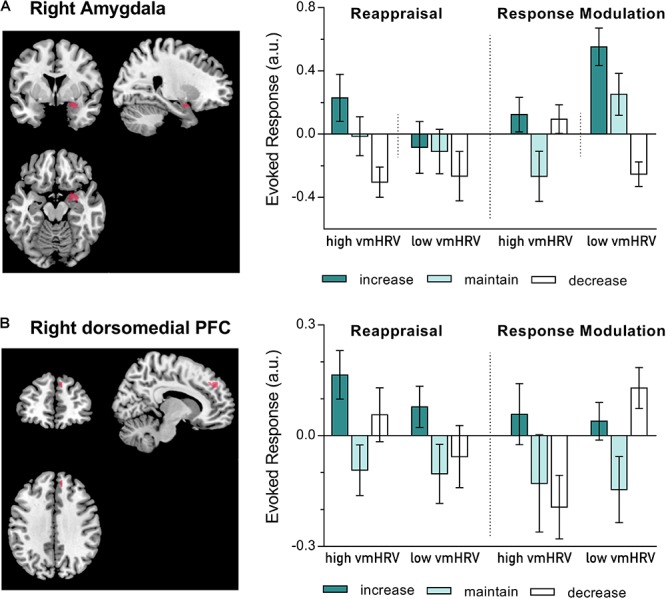
BOLD activation during the regulation of emotions evoked by unpleasant pictures using either reappraisal or response modulation in participants with high and low resting state vagally mediated heart rate variability (vmHRV). On the left side is the BOLD activity at p_uncorr_ = 0_._001, k = 5, overlayed on a standard template (ch2better.nii.gz) using MRIcron (www.cabiatl.com/mricro/mricron). On the right side is the extracted data (individual spheres with a radius of 3 mm). Bars represent group means (arbitrary units) with standard errors. **(A)** Right amygdala activation (y = 2); **(B)** Right dorsomedial prefrontal cortex (PFC) activation (MNI: x = 10–12, y = 44–46, z = 36–38).

Since an influence of resting vmHRV on amygdala activity was only found during regulating emotions evoked by unpleasant pictures, the subsequent analyses of prefrontal regions were conducted for the negative valence category only. Resting vmHRV did not affect responses during ER in the five dorsolateral PFC clusters. In contrast, alike valence ratings and right amygdala responses, responses in the dorsomedial cluster (MNI: x = 12, y = 46, z = 36) during regulating emotions evoked by unpleasant pictures were influenced by resting vmHRV levels as a function of the used regulation strategy (Strategy x Direction x vmHRV: *F*_(2,44)_: 3.59, *p* = .036, *η*^2^_p_ = 0.14; see Figure 3). Compared to the corresponding maintain condition, participants with *high* vmHRV showed enhanced dorsomedial PFC activity while up-regulating unpleasant emotions using reappraisal [*t*_(11)_ = 3.44, *p* = 0.005] but not when using response modulation [*t*_(11)_ = 1.53, *ns*], whereas participants with *low* vmHRV showed no significant difference during reappraisal [*t*_(11)_ = 1.66, *ns*] or response modulation [*t*_(11)_ = 1.78, *ns*]. In contrast, *low* vmHRV participants showed more pronounced dorsomedial PFC activity while down-regulating unpleasant emotions using response modulation [*t*_(11)_ = 2.97, *p* = 0.013] but not while using reappraisal [*t*_(11)_ = 0.39, *ns*], whereas participants with *high* vmHRV showed no significant difference during response modulation [*t*_(11)_ = -0.79, *ns*] or reappraisal [*t*_(11)_ = 1.44, *ns*]. In direct comparison, *low* vmHRV participants showed more pronounced dorsomedial PFC activity while down-regulating unpleasant emotions using response modulation compared to *high* vmHRV participants [*t*_(22)_ = 3.13, *p* = 0.005]. In sum, when regulating unpleasant emotions (mean of dorsomedial activity while up- and down-regulating) compared to maintaining emotions, *high* vmHRV participants showed significantly increased dorsomedial PFC activity when using reappraisal [*t*_(11)_ = 2.38, *p* = 0.037] but not when using response modulation [*t*_(11)_ = 0.70, *ns*]. In contrast, participants with *low* vmHRV showed significantly increased dorsomedial PFC activity when using response modulation [*t*_(11)_ = 2.56, *p* = 0.026] but not when using reappraisal [*t*_(11)_ = 1.10, *ns*] as ER strategy.

## Discussion

This is the first study to combine functional imaging data of the *explicit* ER process and habitual resting state vmHRV data. We observed a differentiated modulation of the activation of the dorsomedial PFC and the amygdala according to the participants’ vmHRV. Thus these results further support recent findings on the relationship between resting state amygdala-PFC functional connectivity and vmHRV, and increase our understanding of the neural concomitants of vmHRV and its role in ER.

We observed that in participants with *high* vmHRV right amygdala activity was modulated according to the regulation direction (i.e., enhanced when instructed to *increase* and diminished when instructed to *decrease*) only when *reappraisal* was used to regulate unpleasant emotions. In contrast, in participants with *low* vmHRV the same modulation of amygdala activity was observed when *response modulation* was used to regulate unpleasant emotions. Similarly, only in participants with *high* vmHRV right dorsomedial PFC activity was increased when using *reappraisal* to regulate unpleasant emotions whereas participants with *low* vmHRV showed increased activation of the right dorsomedial PFC using *response modulation* to regulate unpleasant emotions.

These findings add to the existing findings on the neural concomitants of vmHRV during *implicit* ER (e.g., Lane et al., 2013), and provide further support for the *Neurovisceral Integration Model* (Thayer and Lane, 2000, 2009), which proposes that individuals with high resting state vmHRV are better able to inhibit prepotent emotional responses in the service of more desirable and appropriate ones in accordance with contextual factors. In line with this view, Geisler et al. (2010) have shown that vmHRV is positively associated with subjective well-being, and that this relationship is mediated by the habitual use of executive (i.e., conscious cognitive) ER strategies such as *reappraisal*. Therefore, our data support the findings by Geisler et al. (2010) showing that *high* vmHRV might be beneficial for the *explicit* cognitive regulation of emotions.

Most interestingly, participants with *low* vmHRV showed modulated activation of the right amygdala during the use of *response modulation*. *Response modulation* focuses on changes of respiration, body tension and facial expressions that do not rely on executive (prefrontal) recruitment to the same degree as r*eappraisal,* which involves cognitive variation of the dimension distance-intimacy to the depicted content. Differences in the executive recruitment between those with *low* and *high* vmHRV might explain modulated activation of the amygdala only during the use of *response modulation* in those with *low* vmHRV, while those with *high* vmHRV showed modulated activation of the amygdala during *reappraisal*.

Indeed, our data show that the dorsomedial PFC seems to play an important role in the top-down modulation of the amygdala activation. Whereas only participants with *high* vmHRV showed enhanced activation of the right dorsomedial PFC using *reappraisal* to regulate unpleasant emotions and enhanced modulated activation of the amygdala; participants with *low* vmHRV showed enhanced activation of the right dorsomedial PFC using *response modulation* to regulate unpleasant emotions and enhanced modulated activation of the amgydala. Differences with respect to the ER-direction between individuals with *high* and *low* vmHRV, are potentially related to habitual experiences. Given that lower levels of vmHRV are associated with habitually increased experiences of negative affect (Sloan et al., 2017), the prefrontal cortex results may reflect the greater effort needed to act against one’s habits, i.e., for low vmHRV participants to decrease and for high vmHRV to increase experiences of unpleasant states. Taken together these findings suggest, that those with *low* vmHRV have particular difficulties in conscious, cognitive ER (i.e., *reappraisal*), that involve the recruitment of executive brain areas necessary for the top-down modulation of the amygdala. These findings support the hypothesis that greater resting vmHRV may reflect the efficiency of the prefrontal cortex to regulate amygdala activity in the service of ER.

In line with these observations, we have recently shown that *higher* vmHRV is associated with stronger resting state functional connectivity between the amygdala and the mPFC (Sakaki et al., 2016). Whereas this association was present independent of age, we also found age-related differences in the amygdala’s functional connectivity associated with vmHRV. Compared to older adults, we found that the functional connectivity between the amygdala and the ventrolateral PFC (vlPFC) was more strongly correlated with HRV in younger adults (Sakaki et al., 2016). In line with these findings it has previously been shown that during (*implicit*) ER older and younger adults spontaneously recruit the mPFC (Nashiro et al., 2012), while in addition to the mPFC younger adults also recruit the vlPFC during (*explicit*) ER (Winecoff et al., 2011). Clearly, future studies addressing age related differences in the neural concomitants of *implicit* and *explicit* ER as a function of resting state vmHRV are needed.

Existing research implicates dlPFC regions in voluntary ER and vmPFC regions (including the anterior cingulate cortex, ACC) in automatic – more habitual – ER (Phillips et al., 2008; Silvers et al., 2015). Therefore, it is suggested that *explicit* ER involves greater lateral PFC recruitment (Gyurak et al., 2011), a brain region that shows thinning with increasing age (Fjell et al., 2009). Greater connectivity between vmPFC and amygdala has been shown in older adults even when not told to use *explicit* ER, suggesting that *explicit* ER strategies have become habitual and automatic (Ochsner et al., 2012). Here we have shown in line with a large body of research (Ochsner et al., 2012), that *explicit* ER leads to enhanced activation of the dlPFC. The lack of association of vmHRV with activity of the dlPFC suggests a particular association with vmHRV and *implicit* – automatic – ER. However, we found vmHRV related to activation of the dorsomedial PFC during *explicit* ER. As noted above, this finding suggests that explicit ER strategies that require enhanced recruitment of PFC regions are related to vmHRV. In particular, the present results show that activation of the right dorsomedial PFC significantly varied between participants with *low* and *high* vmHRV according to the ER direction and strategy. Future research is needed to fully explicate the associations of vmHRV with both *implicit* and *explicit* ER.

Differences might also arise as a result of the use of different *explicit* ER strategies. Most fMRI studies on *explicit* ER focused on the down-regulation of unpleasant emotions with *reappraisal* (i.e., the cognitive change of the meaning of a picture; e.g., Ochsner et al., 2002; Eippert et al., 2007; Goldin et al., 2008), or *suppression* (i.e., the inhibition of an ongoing emotion-expressive behavior; e.g., Gross, 1998a,b; Goldin et al., 2008). While our implementation of *reappraisal* is consistent with these prior studies, our implementation of *response modulation* is much broader than that of *suppression*. Here we instructed participants to suppress or enhance their facial expressions or to influence their physiological responses by increasing or decreasing their respiration or their muscle tension. Particularly the regulation of respiration might account for the effectiveness of *response modulation* in participants with *low* vmHRV. Respiration patterns have been shown to reflect the general dimensions of the emotional response (Boiten et al., 1994), in particular the arousal dimension of self-reported emotions (Nyklíček et al., 1997). Previous studies comparing the two ER strategies of *physiological suppression* and *expressive suppression* (Dan-Glauser and Gross, 2011) found that both ER strategies, although targeting different responses, have very similar effects. Therefore, it might be possible that *response modulation* in our study was a multidimensional *explicit* ER strategy, which encompassed the manipulation of one’s facial expression, breathing frequency, and body tension. Future research is needed to clarify whether participants with *low* vmHRV are indeed more effective in non-cognitive ER strategies focusing on manipulations of the breathing frequency.

Finally, the study has several limitations that need to be addressed. vmHRV was recorded using an ambulatory HR monitor (Polar F5 Heart Rate Monitor Watch). Although devices from the respective manufacturer (Polar Electro Oy, Finland) are widely applied for recordings of HRV and show good reliability (e.g., Gamelin et al., 2006; Porto and Junqueira, 2009; Giles et al., 2016), multiple-lead ECG recordings are of better quality and more robust to potential artifacts (i.e., movement). Further, we didn’t implement restrictions regarding physical activity, caffeine intake and smoking prior to recordings of HRV and didn’t control for time of day of the recordings. Future studies, aiming to replicate the present findings, should implement ECG recordings with a more restrictive protocol in the assessment of HRV. We limited our analyses to a time-domain measure of vmHRV (RMSSD), that has been shown to be less affected by respiration and shows greater trait specificity under spontaneous breathing conditions compared to other HRV indices such as spectrally derived high-frequency HRV (Penttilä et al., 2001; Hill et al., 2009; Bertsch et al., 2012). Further, we didn’t control or adjust for levels of mean HR (for a review of the ongoing discussion *see* de Geus et al., 2018).

Finally, the present analyses focused on group differences between individuals with *high* and *low* vmHRV based on a median split. Future studies in larger samples would do well to address a (potential) continuous association between vmHRV and brain function during ER. Such analyses might further address if there are particular levels of vmHRV that predict differences in brain activity during ER. The present approach is justified to a certain degree, given that evidence on altered HRV in individuals with difficulties in ER (i.e., patients with depression), is mainly based on case-control studies – illustrating HRV differences on a group level. It was beyond the scope of the present study to address actual heart-brain interaction during explicit ER. Beyond the present approach, linking resting state vmHRV to brain function during explicit ER, future studies should address how resting vmHRV in turn relates to changes in cardiac activity during ER and how these are related to brain function drawing on recent linear and non-linear techniques for the assessment of heart-brain coupling (e.g., Valenza et al., 2016).

With respect to the fMRI assessments, scanner noise may impact affective states (i.e., Jacob et al., 2015). Similar to other research in the domain, the present study faces these challenges. However, experimental conditions between groups based on vmHRV were identical. Thus, it is unlikely that scanner noise had an impact on the present findings. vmHRV differs by sex (Koenig and Thayer, 2016), such that females show greater vmHRV compared to males. Future studies should address potential sex differences in the association between vmHRV and neural activity during ER, as the present sample was too small to investigate this factor.

### Summary

In conclusion, our results replicate and extend findings from neuroimaging studies on ER and significantly add to the literature by showing that different levels of resting state vmHRV predict different patterns of neural activity during *explicit* ER. In line with the process model of ER (Gross, 1998b), we observed enhanced activation of prefrontal brain structures associated with emotional control whereas activation of the amygdala was modulated according to the ER direction. Our data provide first evidence for neural differences during explicit ER using different ER strategies as a function of resting state vmHRV. Participants with *high* vmHRV only show modulated activation of the right amygdala during the use of *reappraisal* whereas participants with *low* vmHRV show modulated activation of the right amygdala only during the use of *response modulation.* Further, we found increased activation of the dorsomedial PFC in participants with *high* vmHRV when regulating unpleasant emotions with reappraisal and in participants with *low* vmHRV when regulating unpleasant emotions when using response modulation. Future studies need to address age related differences in the neural concomitants of vmHRV during *explicit* ER and should aim to replicate these findings in samples with difficulties in ER (i.e., depressed patients). As habitual ER strategies have been shown to be related to vmHRV (Geisler et al., 2010) and brain activity when viewing emotional stimuli (e.g., Abler et al., 2010), future studies should also address how habitual ER strategies relate to the present findings. Adding to existing findings on the neural concomitants of vmHRV during *implicit* ER, the present findings support the *Neurovisceral Integration Model* (Thayer and Lane, 2000, 2009), suggesting that higher vmHRV is associated with neural mechanisms that support successful ER.

## Author Contributions

ES, JW, and FG were involved in data collection, analysis and writing of the manuscript. AH and JT contributed to the critical design of the study. JK and JW wrote the first draft of the manuscript. All authors provided important intellectual content in revising the manuscript and approved the final version before submission.

## Conflict of Interest Statement

The authors declare that the research was conducted in the absence of any commercial or financial relationships that could be construed as a potential conflict of interest.

## Footnotes

1 The overall results of this analysis are reported in Steinfurth et al. (2013).

2 The following randomized orders were generated: Order one started with a pleasant picture and the instruction to increase, order two started with a pleasant picture and the instruction to just view passively, order three started with a pleasant picture and the instruction to decrease the emotion, order four started with an unpleasant picture and the instruction to just view passively, order five started with an unpleasant picture and the instruction to decrease the appearing emotion and order six started with an unpleasant picture and the instruction to increase the emotion.
