# Supplementary material for: Resting State Vagally-Mediated Heart Rate Variability Is Associated With Neural Activity During Explicit Emotion Regulation
Source: Front Neurosci. 2018 Nov 5;12:794. doi: 10.3389/fnins.2018.00794 (PMC6231057; doi:10.3389/fnins.2018.00794)
Supplement: Supplementary file 1 [file Table_1.DOCX]

**Supplementary Table 1**. Results of repeated-measures ANOVAs for valance ratings, right amygdala and dorsomedial prefrontal cortex responses.

| **Source** | **df** | **MS** | **F** | **p** | **Effect Size** |
| --- | --- | --- | --- | --- | --- |
| ***Valence ratings*** | | | | | |
| Strategy | 1 | .731 | 9.291 | .006 | .297 |
| Strategy × vmHRV | 1 | .338 | 4.298 | .050 | .163 |
| Error (Strategy) | 22 | .079 |  |  |  |
| Valence | 1 | 15.620 | 18.545 | .000 | .457 |
| Valence × vmHRV | 1 | 2.526 | 2.999 | .097 | .120 |
| Error (Valence) | 22 | .842 |  |  |  |
| Direction | 2 | .066 | .960 | .391 | .042 |
| Direction × vmHRV | 2 | .026 | .379 | .687 | .017 |
| Error (Direction) | 44 | .068 |  |  |  |
| Strategy × Valence | 1 | .384 | 3.704 | .067 | .144 |
| Strategy × Valence × vmHRV | 1 | .359 | 3.461 | .076 | .136 |
| Error (Strategy × Valence) | 22 | .104 |  |  |  |
| Strategy × Direction | 2 | .179 | 2.760 | .074 | .111 |
| Strategy × Direction × vmHRV | 2 | .130 | 2.004 | .147 | .083 |
| Error (Strategy × Direction) | 44 | .065 |  |  |  |
| Valence × Direction* | 1.365 | 1.435 | 5.820 | .014 | .209 |
| Valence × Direction × vmHRV* | 1.365 | .042 | .168 | .761 | .008 |
| Error (Valence × Direction) | 44 | .168 |  |  |  |
| Strategy × Valence × Direction* | 1.600 | .393 | 4.921 | .019 | .183 |
| Strategy × Valence × Direction × vmHRV* | 1.600 | .381 | 4.770 | .021 | .178 |
| Error (Strategy × Valence × Direction) | 44 | .064 |  |  |  |
| ***Right amygdala responses*** | | | | | |
| Strategy | 1 | .831 | 2.999 | .097 | .120 |
| Strategy × vmHRV | 1 | .722 | 2.608 | .121 | .106 |
| Error (Strategy) | 22 | .277 |  |  |  |
| Valence | 1 | .054 | .263 | .613 | .012 |
| Valence × vmHRV | 1 | .076 | .370 | .549 | .017 |
| Error (Valence) | 22 | .206 |  |  |  |
| Direction | 2 | 3.137 | 9.618 | .000 | .304 |
| Direction × vmHRV | 2 | .126 | .388 | .681 | .017 |
| Error (Direction) | 44 | .326 |  |  |  |
| Strategy × Valence | 1 | .327 | 1.263 | .273 | .054 |
| Strategy × Valence × vmHRV | 1 | .278 | 1.073 | .312 | .046 |
| Error (Strategy × Valence) | 22 | .259 |  |  |  |
| Strategy × Direction | 2 | .297 | 2.105 | .134 | .087 |
| Strategy × Direction × vmHRV | 2 | .756 | 5.357 | .008 | .196 |
| Error (Strategy × Direction) | 44 | .141 |  |  |  |
| Valence × Direction | 2 | .022 | .172 | .843 | .008 |
| Valence × Direction × vmHRV | 2 | .689 | 5.286 | .009 | .194 |
| Error (Valence × Direction) | 44 | .130 |  |  |  |
| Strategy × Valence × Direction | 2 | .014 | .079 | .924 | .004 |
| Strategy × Valence × Direction × vmHRV | 2 | .533 | 3.114 | .054 | .124 |
| Error (Strategy × Valence × Direction) | 44 | .171 |  |  |  |
| ***Dorsomedial prefrontal cortex responses*** | | | | | |
| Strategy | 1 | .082 | .727 | .403 | .032 |
| Strategy × vmHRV | 1 | .244 | 2.180 | .154 | .090 |
| Error (Strategy) | 22 | .112 |  |  |  |
| Direction | 2 | .493 | 7.342 | .002 | .250 |
| Direction × vmHRV | 2 | .079 | 1.174 | .318 | .051 |
| Error (Direction) | 44 | .067 |  |  |  |
| Strategy × Direction | 2 | .006 | .128 | .880 | .006 |
| Strategy × Direction × vmHRV | 2 | .165 | 3.589 | .036 | .140 |
| Error (Strategy × Direction) | 44 | .046 |  |  |  |

*Greenhouse-Geisser corrected for violation of sphericity; df = degrees of freedom; MS = mean squares, Effect size = partial eta-squared ($\eta_{p}^{2}$).
